# Neurochondrin interacts with the SMN protein suggesting a novel mechanism for spinal muscular atrophy pathology

**DOI:** 10.1242/jcs.211482

**Published:** 2018-04-17

**Authors:** Luke W. Thompson, Kim D. Morrison, Sally L. Shirran, Ewout J. N. Groen, Thomas H. Gillingwater, Catherine H. Botting, Judith E. Sleeman

**Affiliations:** 1 School of Biology, University of St Andrews, BSRC Complex, North Haugh St Andrews, KY16 9ST, UK; 2 Edinburgh Medical School, Biomedical Sciences and Euan MacDonald Centre for Motor Neuron Disease Research, University of Edinburgh, Hugh Robson Building, George Square, Edinburgh, EH8 9XD, UK

**Keywords:** Spinal muscular atrophy, Neurochondrin, SMN1, SMN2, SmB, SmN, Sm protein, snRNP

## Abstract

Spinal muscular atrophy (SMA) is an inherited neurodegenerative condition caused by a reduction in the amount of functional survival motor neuron (SMN) protein. SMN has been implicated in transport of mRNA in neural cells for local translation. We previously identified microtubule-dependent mobile vesicles rich in SMN and SNRPB, a member of the Sm family of small nuclear ribonucleoprotein (snRNP)-associated proteins, in neural cells. By comparing the interactomes of SNRPB and SNRPN, a neural-specific Sm protein, we now show that the essential neural protein neurochondrin (NCDN) interacts with Sm proteins and SMN in the context of mobile vesicles in neurites. NCDN has roles in protein localisation in neural cells and in maintenance of cell polarity. NCDN is required for the correct localisation of SMN, suggesting they may both be required for formation and transport of trafficking vesicles. NCDN may have potential as a therapeutic target for SMA together with, or in place of the targeting of SMN expression.

This article has an associated First Person interview with the first author of the paper.

## Abstract (precis)

**Highlighted Article:** The essential neural protein neurochondrin interacts with the spinal muscular atrophy (SMA) protein SMN in cell lines and in mice. This might be relevant to the molecular pathology of SMA.

## INTRODUCTION

The inherited neurodegenerative disease spinal muscular atrophy (SMA) is caused by a reduction in the amount of functional survival motor neuron (SMN) protein (Lefebvre et al., 1995). SMA is the leading genetic cause of infant mortality, affecting 1:6000 live births (Monani, 2005). The recently developed therapy, treatment with Spinraza/Nusinersen (Biogen), has been shown to increase the level of SMN and improve the symptoms of SMA patients (Corey, 2017; Finkel et al., 2016; Passini et al., 2011). Most SMA patients harbour mutations in the *SMN1* gene, which produces the majority of total SMN protein in cells. In humans, expression from a variable number of copies of an additional gene, *SMN2*, can produce some full-length SMN protein (Lefebvre et al., 1995, 1997). The SMN2 gene, unlike SMN1, contains a point mutation in an exon splicing enhancer (Lorson and Androphy, 2000; Lorson et al., 1999) resulting in truncation of most of the SMN protein produced by SMN2 through skipping of exon 7. The truncated protein produced by *SMN2* is less stable than full-length SMN and cannot compensate fully for the loss of *SMN1* (Le et al., 2005; Lorson and Androphy, 2000; Lorson et al., 1999). However, owing to the small amounts of full-length SMN expressed from the *SMN2* gene, the number of gene copies can influence the severity of SMA, with evidence that five copies of *SMN2* may be enough to compensate for loss of *SMN1* (Campbell et al., 1997; Prior et al., 2004). It is not currently clear how a deficiency of functional SMN leads to the specific symptoms of SMA. In particular, the differing sensitivity of cell types to lowered SMN levels, with motor neurons most severely affected, is difficult to explain as SMN is an essential protein and complete deletion is lethal at the cellular level (Hsieh-Li et al., 2000; Schrank et al., 1997).

SMN localises to nuclear Cajal bodies (CBs) and gemini of Cajal bodies (gems) (Liu and Dreyfuss, 1996) as well as in the cytoplasm, and is implicated in a growing number of cellular roles in both locations (Hosseinibarkooie et al., 2017; Li et al., 2014; Monani, 2005; Singh et al., 2017; Sleeman, 2013; Tisdale and Pellizzoni, 2015). The first role to be elucidated was a role in the early, cytoplasmic, stages of assembly and maturation of splicing small nuclear ribonucleoproteins (snRNPs). Splicing snRNPs are ribonucleoprotein complexes that are essential for pre-mRNA splicing, and comprise a small nuclear RNA (snRNA) core and numerous proteins, including a heptameric ring containing one copy each of members of the Sm protein family. SMN is part of a cytoplasmic complex, also containing the gemin proteins that is required for the addition of the Sm proteins as a ring around the snRNA core (Li et al., 2014; Liu et al., 1997; Stark et al., 2001; Tisdale and Pellizzoni, 2015). The maturation of snRNPs has been shown to be impaired by a deletion in SMN (Gabanella et al., 2007; Shpargel and Matera, 2005; Wan et al., 2005; Winkler et al., 2005; Zhang et al., 2008), while alterations to pre-mRNA splicing events, proposed to be a downstream consequence of this impairment, have been observed in several models of SMA (Custer et al., 2013; Huo et al., 2014; Zhang et al., 2008). One of the proposed mechanisms for the cell-type specificity of SMA is that these alterations of pre-mRNA splicing events affect mRNA transcripts that are essential for motor neurons, perhaps preferentially affecting transcripts spliced by the minor spliceosome (Boulisfane et al., 2011; Custer et al., 2016; Doktor et al., 2017; Gabanella et al., 2007; Zhang et al., 2008). Despite promising results in *Drosophila* models, however, specific transcripts affecting motor neurons are yet to be conclusively identified (Lotti et al., 2012).

Another well-established cellular role of SMN is in the trafficking of mature mRNA within the cytoplasm, particularly in the axons and neurites of neural cell types (Akten et al., 2011; Custer et al., 2013; Fallini et al., 2016, 2014, 2011; Li et al., 2015; Lotti et al., 2012; Peter et al., 2011; Rossoll et al., 2003, 2002; Todd et al., 2010a,b; Zhang et al., 2006, 2003). This is thought to be linked to local translation of mRNA into proteins, an important process for neural cells, in particular motor neurons, owing to the length of their axons (Doyle and Kiebler, 2011; Holt and Schuman, 2013; Huber et al., 2000; Kang and Schuman, 1996), making this trafficking role for SMN of particular interest for understanding the cellular pathology of SMA. SMN colocalises with the mRNA-binding proteins HuD (also known as ELAVL4), IMP1 (also known as IGF2BP1) and heterogeneous nuclear RNP (hnRNP) R and is involved in the localisation of mRNAs to axons (Akten et al., 2011; Fallini et al., 2014, 2011; Rossoll et al., 2003). The cellular structures involved in SMN-dependent mRNA trafficking are currently unclear, being described as granular (Akten et al., 2011; Fallini et al., 2014; Peter et al., 2011; Todd et al., 2010b; Zhang et al., 2006, 2003) or vesicular in nature (Custer et al., 2013; Prescott et al., 2014).

SMN has also been implicated in many other processes. Some of these involve a role in RNP assembly, similar to the canonical role in splicing snRNP production, including assembly of both the signal recognition particle and the U7 snRNP required for 3′ processing of histone mRNA (Azzouz et al., 2005; Piazzon et al., 2013; Tisdale et al., 2013). Other roles are more diverse, and include the regulation of cytoskeletal dynamics and endocytosis (Bowerman et al., 2007; Dimitriadi et al., 2016; Giesemann et al., 1999; Hao le et al., 2012; Heesen et al., 2016; Hosseinibarkooie et al., 2016; Nölle et al., 2011; Oprea et al., 2008; Riessland et al., 2017), enhancement of DNA repair (Takaku et al., 2011); transcriptional regulation (Pellizzoni et al., 2001; Zhao et al., 2016; Zou et al., 2004), stress granule formation (Hua and Zhou, 2004; Zou et al., 2011) and ubiquitin homeostasis (Wishart et al., 2014). It is currently unclear whether or how disruption of the many proposed roles for SMN contributes to SMA pathogenesis.

Structures containing both the SMN and SNRPB (also known as SmB) proteins, alongside coatomer γ are trafficked on microtubules (Prescott et al., 2014). Evidence suggesting that SMN- and SNRPB-rich structures are vesicular in nature includes their staining with lipophilic dyes in living neural cells, their COPI vesicular appearance as seen with correlative fluorescence and electron microscopy (Prescott et al., 2014), and the interaction of SMN and the Sm proteins with coatomer proteins, which are associated with membrane-bound COPI vesicles (Custer et al., 2013; Peter et al., 2011; Prescott et al., 2014). We have previously identified an interaction between SNRPB and dynein cytoplasmic 1 heavy chain 1 (DYNC1H1), a motor protein required for microtubule transport (Prescott et al., 2014), mutation in which can cause a rare, lower-extremity dominant SMA (Chen et al., 2017; Ding et al., 2016; Harms et al., 2010, 2012; Niu et al., 2015; Peeters et al., 2015; Punetha et al., 2015; Scoto et al., 2015; Strickland et al., 2015; Tsurusaki et al., 2012). SMN has also been shown to associate with the membranous Golgi complex (Ting et al., 2012), while mutations in the Golgi-related protein bicaudal D homolog 2 (BICD2) also cause a form of lower-extremity dominant SMA (Martinez-Carrera and Wirth, 2015; Neveling et al., 2013; Oates et al., 2013; Peeters et al., 2013; Rossor et al., 2015; Synofzik et al., 2014).

The Sm protein family is implicated in both of the major functions of SMN. The core of splicing snRNPs comprises a heptameric ring of proteins around the snRNA, containing SNRPB and/or its alternatively spliced variant SNRPB′, SNRPD1, SNRPD2, SNRPD3, SNRPE, SNRPF and SNRPG (Urlaub et al., 2001). SMN is a vital part of the complex required for the assembly of this Sm protein ring (Battle et al., 2006; Fischer et al., 2011, 1997; Liu and Dreyfuss, 1996; Meister et al., 2001; Meister and Fischer, 2002; Pellizzoni et al., 2002). Other members of the Sm protein family have also been identified, beyond the core proteins usually found in splicing snRNPs. Of particular interest in the context of SMA pathology is SNRPN (this protein is also known as SmN, denoted with a lowercase ‘m’), which is expressed in neural tissues (Schmauss et al., 1992) and can replace SNRPB in the heptameric Sm protein ring (Huntriss et al., 1993). The human SNRPN protein differs from SNRPB′ by 17 amino acids (UniProt identifiers P63162 and P14678, respectively), and little is known about its behaviour other than its incorporation into snRNPs, although the *SNRPN* gene locus is within the paternally imprinted region of the genome critical in Prader–Willi syndrome (Özçelik et al., 1992). There is growing appreciation that some Sm proteins may ‘moonlight’ in functions beyond their presence in splicing snRNPs. In addition to the role of SNRPB in cytoplasmic trafficking vesicles in human cells, in *Drosophila* the homologues of SNRPB and SNRPD3 are implicated in mRNA localisation (Gonsalvez et al., 2010) and SNRPD1 has a role in micro (mi)RNA biogenesis (Xiong et al., 2015). With this in mind, we applied a proteomic approach in the neural cell line SH-SY5Y to search for interactions that could indicate neural-specific roles for the SNRPN protein that are of relevance for the pathology of SMA.

This proteomic approach led to the identification of neurochondrin (NCDN) as a novel interactor of both SNRPN and SMN. NCDN is an essential protein that is predominantly expressed in neural tissue, and is involved in neural outgrowth, synaptic plasticity and moderation of signal transduction (Dateki et al., 2005; Francke et al., 2006; Matosin et al., 2015; Pan et al., 2016; Shinozaki et al., 1999, 1997; Wang et al., 2013, 2009; Ward et al., 2009). Further investigation of the relationship between NCDN and SMN suggests that NCDN interacts with SMN in the context of mobile cytoplasmic vesicles containing SNRPB and SNRPN and is strongly expressed in motor neurons. This suggests that NCDN warrants further investigation in the context of SMA pathology and that it might prove useful as a target for future therapy development.

## RESULTS

### SNRPN exhibits similar behaviour to SNRPB, localising to cytoplasmic vesicles containing SMN

**Fig. 1.**
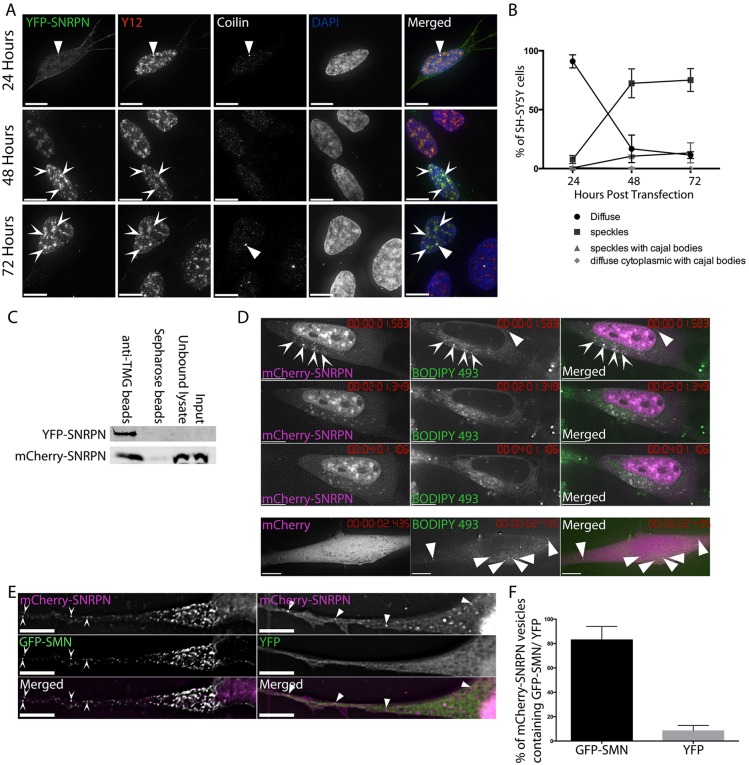
**SNRPN exhibits similar behaviour** **to SNRPB in SH-SY5Y cells.** (A) SH-SY5Y cells transiently expressing YFP–SNRPN and fixed after 24, 48 and 72 h show variations in distribution of the YFP–SNRPN with time. Immunostaining with Y12 (detecting Sm proteins, red on overlay) and anti-coilin (white on overlay) shows splicing speckles (chevron arrowheads) and cajal bodies (CBs, triangular arrowheads) respectively. Images are deconvolved *z*-stacks with 0.2 µm spacing. (B) After transient expression, SNRPN initially localises diffusely in the cytoplasm, before localising to speckles at the 48 and 72 h time-points. Results are mean±s.d. from three independent experiments, *n*=100 cells per experiment. (C) Western blot analysis of snRNPs immunoprecipitated using TMG beads (against the characteristic tri-methyl guanosine Cap of snRNAs, left hand lane) confirms that both YFP–SNRPN (detected with anti-YFP, top row) and mCherry–SNRPN (detected with anti-mCherry, bottom row) are incorporated into snRNPs. (D) mCherry–SNRPN cytoplasmic structures are mobile and stain with the lipophilic dye BODIPY 493. Chevron arrowheads identify mCherry–SNRPN structures stained with BODIPY 493; triangular arrowheads identify BODIPY 493-stained vesicles not containing mCherry–SNRPN. mCherry alone does not accumulate in BODIPY 493-stained vesicles. Cells were imaged approximately every 4 s for 9 min. Images are single deconvolved *z*-sections. (E) mCherry–SNRPN and GFP–SMN colocalise in cytoplasmic foci in SH-SY5Y cells (chevron arrowheads in left hand panels), whereas YFP alone shows no accumulation in mCherry-SNRPN foci (triangular arrowheads in right hand panels). White signal on the overlay indicates areas of colocalisation. Images are single deconvolved *z*-sections. (F) Comparison of the percentage of mCherry–SNRPN vesicles per cell colocalising with GFP–SMN to those showing co-incidental overlap with YFP alone confirms the colocalisation. Results are mean±s.d., *n*=5 (*P*<0.0001, unpaired two-tailed *t*-test). Scale bars: 7 µm.

In order to determine the interactome of the neural-specific Sm protein SNRPN, we first generated constructs to express fluorescent protein-tagged SNRPN by amplifying the SNRPN sequence from total RNA obtained from SH-SY5Y cells and cloning it into the pEYFP-C1 and pmCherry-C1 vectors. All of the Sm fusion proteins studied so far show a steady-state localisation to nuclear CBs and speckles. The Sm proteins SNRPB, SNRPD1 and SNRPE have also previously been shown to exhibit a characteristic pathway within the cell on their initial expression, indicative of the snRNP maturation pathway (Sleeman and Lamond, 1999), although differences were seen between the Sm proteins. To confirm that YFP–SNRPN localised correctly at steady state to CBs and speckles, and to determine where SNRPN localised during maturation and incorporation into snRNPs, the plasmid was transiently expressed in SH-SY5Y neuroblastoma cells, with cells fixed and immunostained at 24 h intervals. At 48 and 72 h after transfection, YFP–SNRPN predominantly localised to speckles and CBs (as detected with anti-coilin antibody) identically to endogenous Sm proteins (detected with Y12 antibody), whereas at 24 h, YFP–SNRPN localised predominantly diffusely within the cytoplasm, with some accumulation in CBs (Fig. 1A,B). This sequential localisation is indistinguishable from that previously observed with YFP–SNRPB in HeLa and MCF-7 cells, though CBs were not prominent in the majority of SH-SY5Y cells transiently expressing YFP–SNRPN. Equivalent results were also obtained in SH-SY5Y cells transiently expressing mCherry–SNRPN (Fig. S1). Both YFP–SNRPN and mCherry–SNRPN are efficiently incorporated into splicing snRNPs, as evidenced by their enrichment from whole-cell lysates when using antibodies against the characteristic hypermethylated Cap structure (2,2,7-trimethylaguanosine) found on snRNAs (Fig. 1C).

To determine whether the similarities between SNRPN and SNRPB extend to their localisation in detergent-sensitive vesicles in the cytoplasm (Prescott et al., 2014), SH-SY5Y cells constitutively expressing mCherry–SNRPN were used for live-cell time-lapse microscopy. Mobile mCherry–SNRPN foci were observed (Movie 1). In common with the SNRPB vesicles, these stained positive with the lipophilic dye BODIPY 493, indicating that they are vesicular in nature (Fig. 1D). Finally, to confirm that the mCherry–SNRPN vesicles were similar to those previously identified with SNRPB, SH-SY5Y cells constitutively expressing mCherry–SNRPN were transfected with plasmids to express either GFP–SMN or YFP alone. GFP–SMN colocalised with mCherry–SNRPN in 83% (±11, s.d.) of SNRPN-positive vesicles, which is statistically significant when compared to 8.4% (±4.5) of mCherry–SNRPN vesicles colocalising with YFP alone (Fig. 1E,F).

### Mass spectrometry reveals similarities between the interactomes of SNRPN and SNRPB

**Fig. 2.**
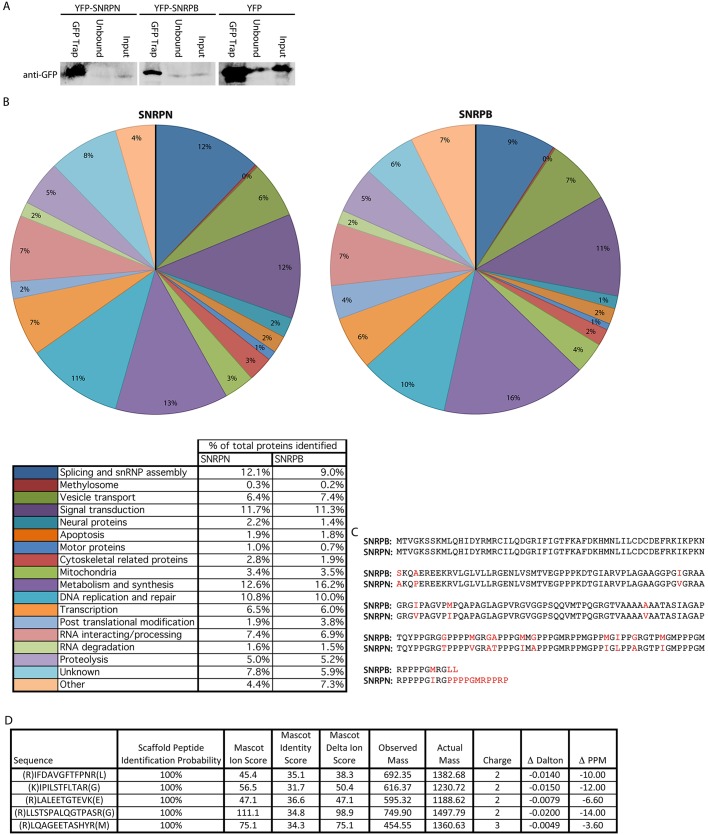
**The interactomes of SNRPB and SNRPN are similar, but there are differences at the level of individual proteins.** (A) Immunoblot analysis confirms efficient affinity purification of YFP–SNRPN, YFP–SNRPB and YFP. 10% of the affinity purified material (left hand lane in each panel) was compared to 80 µg of precleared lysate (Input) and unbound material using anti-GFP antibody. GFP-Trap effectively immunoprecipitated all three proteins. (B) After processing the MS data and sorting identified proteins into groups based on Gene Ontology annotations, the interactomes of SNRPN and SNRPB are very similar. (C) A comparison between the amino acid sequences of SNRPN and SNRPB reveals their similarity. Differences in amino acid sequence are in red. Sequences are from Uniprot [entries P63162 (SNRPN) and P14678-2 (SNRPB)]. (D) NCDN was identified in the interactome of YFP–SNRPN, with five unique peptide hits encompassing 9% sequence coverage. Each Ion score (Mascot Ion Score) was above the threshold for peptide identity (Mascot Identity Score), with two out of the five identified peptides having a score of above double the threshold score.

As SNRPN appeared to behave very similarly to SNRPB in neural cells, it was unclear why neural cells express two almost identical proteins. We decided to investigate whether SNRPN and SNRPB may have differing roles that could be identified by proteomic analysis. SH-SY5Y cells were selected for this analysis as they are easy to culture and amenable to the generation of cell lines constitutively expressing fluorescent protein-tagged proteins, while retaining neural characteristics including the expression of neural proteins. They are also human in origin. Proteins interacting with YFP–SNRPB and YFP–SNRPN were affinity purified by means of GFP-TRAP (Chromotek) from whole-cell lysates of SH-SY5Y cell lines constitutively expressing the tagged proteins, with a cell line expressing YFP alone as a control for non-specific binding to the tag or bead matrix. Immunoblot analysis using antibodies to YFP demonstrated that the enrichment of the tagged proteins was 20×, 23× and 4× for YFP–SNRPN, YFP–SNRPB and YFP alone, respectively (Fig. 2A). The affinity purified material was size separated by SDS-PAGE and analysed by nano-liquid chromatography electrospray ionisation tandem mass spectrometry (nLC ESI MS/MS) to identify peptides and, hence, proteins interacting with YFP–SNRPB and YFP–SNRPN. Following removal of likely contaminants identified by their interaction with YFP alone, or their previous identification as common contaminants in GFP-TRAP experiments (Trinkle-Mulcahy et al., 2008), UniProt Biological Process and Cellular Component Genome Ontology annotations were used to group identified proteins into categories depending on function. These groups were then used to determine whether there were differences in possible functions between SNRPN and SNRPB (Fig. 2B) despite their similarity (Fig. 2C). Numerous proteins previously established to interact with Sm proteins were identified, including SMN and the gemins as well as the methylosome components PRMT5, MEP50 (also known as WDR77) and pICIn (also known as CLNS1A), validating our approach (Table S1). The overall proportions of proteins in each category were similar when comparing the interactomes of SNRPN to SNRPB, although differences were identified at the level of individual proteins. Of particular interest in the context of SMA were a number of proteins with potential neural-specific roles, which were identified in one or both samples. One of these was NCDN, a relatively poorly characterised neural protein, which was identified in the YFP–SNRPN interactome, with five unique peptides identified (Fig. 2D).

### Neurochondrin interacts with SNRPN, SNRPB and SMN in cell lines and *in vivo*

**Fig. 3.**
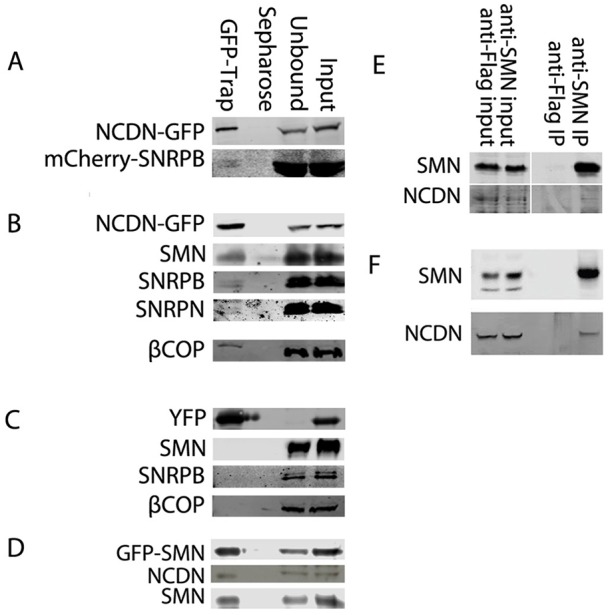
**NCDN interacts with SNRPN, SNRPB and SMN in cell lines and in mice.** (A) Affinity isolation of NCDN–GFP using GFP-Trap, detected with anti-GFP antibody (top row) co-enriches mCherry–SNRPB, detected with anti-mCherry (bottom row) in transiently co-transfected SH-SY5Y cells. (B) In an SH-SY5Y cell line constitutively expressing NCDN–GFP, affinity isolation of NCDN–GFP, detected with anti-GFP antibody (top row) co-enriches SMN, SNRPB, SNRPN and the coatomer protein βCOP, all detected with antibodies against the endogenous proteins (as labelled). (C) In an SH-SY5Y cell line constitutively expressing YFP, affinity isolation of YFP, detected with anti-GFP antibody (top row) does not co-enrich SMN, SNRPB or βCOP, all detected with antibodies to the endogenous proteins (as labelled). (D) In an SH-SY5Y cell line constitutively expressing GFP–SMN, affinity isolation of GFP–SMN, detected with anti-GFP antibody (top row) co-enriches endogenous NCDN, detected with anti-NCDN antibody (middle row). Endogenous SMN, detected with anti-SMN (bottom row) is also co-enriched. (E) Immunoprecipitation (IP) of endogenous SMN co-enriches endogenous NCDN in SH-SY5Y cells. (F) Immunoprecipitation of endogenous SMN from murine P8 brain lysate co-enriches NCDN.

To verify the interaction between Sm proteins and NCDN, a construct expressing NCDN–GFP was generated. Affinity purification of NCDN–GFP from whole-cell lysates of SH-SY5Y cells transiently co-expressing NCDN–GFP and mCherry–SNRPB demonstrated interaction between NCDN–GFP and mCherry–SNRPB (Fig. 3A). To further investigate interactions between NCDN and the Sm proteins in neural cells, an SH-SY5Y cell line constitutively expressing NCDN–GFP was established. Affinity purification of NCDN–GFP from whole-cell lysates followed by immunoblot analysis using antibodies against endogenous SNRPN and SNRPB (Fig. 3B) revealed that NCDN–GFP interacts with both SNRPN and SNRPB. Furthermore, both endogenous SMN and endogenous βCOP (a coatomer vesicle protein; also known as COPB1) were also revealed to interact with NCDN–GFP, suggesting that NCDN interacts with the Sm proteins and SMN in the context of cytoplasmic vesicles. Affinity purification of YFP alone from whole-cell lysates of an SH-SY5Y cell line constitutively expressing YFP does not result in co-purification of endogenous SMN, SNRPB or βCOP (Fig. 3C). To further investigate the interaction between SMN and NCDN observed in Fig. 3B, a reciprocal experiment was performed using GFP-TRAP to affinity purify GFP–SMN from an SH-SY5Y cell line constitutively expressing GFP–SMN (Clelland et al., 2009). Subsequent immunoblot analysis using antibodies to endogenous NCDN demonstrated that NCDN co-enriched with GFP–SMN (Fig. 3D). To determine whether this interaction also occurs between the endogenous proteins at normal expression levels, we first immunoprecipitated endogenous SMN from whole-cell lysates of SH-SY5Y cells. Immunoblot analysis using antibodies against endogenous NCDN (Fig. 3E) confirmed that endogenous SMN interacts with endogenous NCDN. Moreover, to determine whether this interaction is also present at endogenous levels *in vivo*, and thus of potential relevance to SMA pathology, SMN was immuno-precipitated from lysates of post-natal day (P)8 mouse brain. Again, we confirmed that endogenous SMN interacts with endogenous NCDN *in vivo* (Fig. 3F). Taken together, these results confirm the interaction of SMN and NCDN at endogenous levels and *in vivo*.

### Neurochondrin colocalises with SNRPN, SNRPB and SMN in cytoplasmic vesicles but not nuclear foci and is strongly expressed in motor neurons in mouse spinal cord

**Fig. 4.**
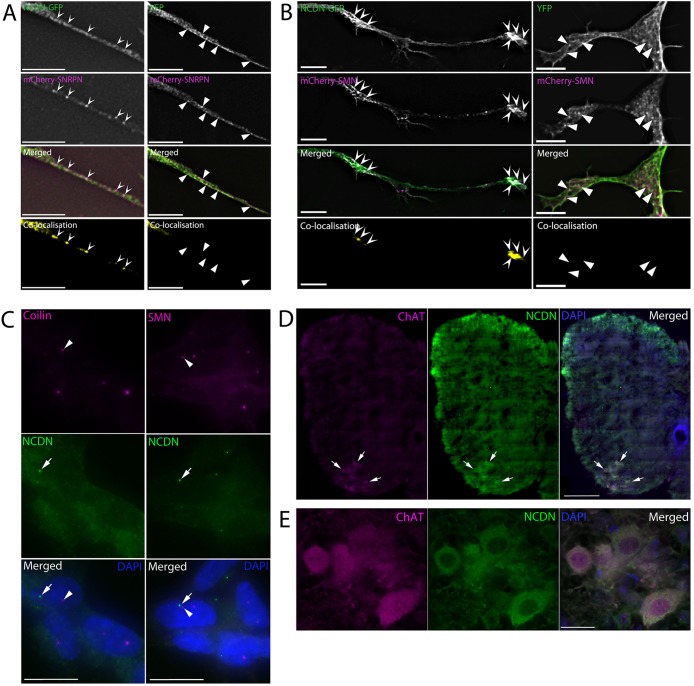
**NCDN colocalises with SNRPN and SMN in the cytoplasm, but not the nucleus, and is expressed in motor neurons in mouse spinal cord.** (A) NCDN–GFP and mCherry–SNRPN colocalise in vesicle-like structures (chevron arrowheads) in neurites of SH-SY5Y cells constitutively expressing mCherry–SNRPN, and transiently expressing NCDN-GFP (left hand panels). White areas in the merged image show areas of colocalisation. Colocalisation images (bottom row) were generated in Volocity, using automatic thresholds on undeconvolved z-sections before excluding values below 0.05 (see Materials and Methods). No colocalisation is seen in the same cell line transiently expressing YFP alone (right hand panel). Triangular arrowheads show structures containing mCherry–SNRPN but not YFP. (B) mCherry–SMN and NCDN–GFP colocalise in vesicles (chevron arrowheads) in the cytoplasm of co-transfected SH-SY5Y cells (left hand panels). White areas in the merged image show areas of colocalisation. Colocalisation images (bottom row) were generated as above. No colocalisation is observed between mCherry-SMN and YFP (triangular arrowheads, right hand panels). (C) NCDN forms nuclear foci (arrows) in the nuclei of a small proportion of SH-SY5Y cells (≤2%, two independent experiments, *n*=100 cells per experiment). These do not colocalise with nuclear foci stained with coilin (arrowheads, left hand panels) or SMN (arrowheads, right hand panels). (D) NCDN (green) is expressed throughout the spinal cord, with increased expression in motor neurons (arrows), as identified with anti-ChAT antibody (magenta). (E) Higher magnification imaging confirms the presence of NCDN in ChAT-positive motor neurons (single deconvolved z-section). Scale bars: 7 µm (A,B); 500 µm (C,D); 10 µm (E).

To determine the probable cellular location for the interaction between SNRPN or SNRPB and NCDN, plasmids to express either NCDN–GFP or YFP were transiently transfected into SH-SY5Y cells constitutively expressing mCherry–SNRPN. This revealed that NCDN–GFP, but not YFP alone, accumulates in cytoplasmic vesicles containing mCherry–SNRPN (Fig. 4A). Similar results were obtained when NCDN–GFP was transiently expressed in SH-SY5Y cells constitutively expressing mCherry–SNRPB (Fig. S2). To investigate the probable cellular location of interactions between SMN and NCDN, mCherry–SMN was co-expressed with either NCDN–GFP or YFP alone. NCDN–GFP was found in cytoplasmic structures enriched in mCherry–SMN (Fig. 4B). These data suggest that NCDN colocalises with both the Sm proteins and SMN in cytoplasmic vesicles, although NCDN–GFP has a higher level of diffuse signal than the Sm proteins or SMN. Within the nucleus, antibodies to endogenous NCDN showed very little nuclear staining, with nuclear foci evident in very few cells (≤2%). These foci did not stain with antibodies to either coilin or SMN (Fig. 4C) indicating that they are neither CBs nor gems. In sections from murine P5 spinal cord (Fig. 4D,E), NCDN shows robust expression throughout the spinal cord. Interestingly, NCDN was most prominently expressed in choline acetyltransferase (ChAT)-positive motor neurons in the ventral horn of the spinal cord (arrows in Fig. 4D, enlarged in Fig. 4E). This indicates that NCDN is enriched in motor neurons: the most relevant cell type for SMA.

### NCDN, SMN and Sm proteins co-fractionate with coatomer proteins

**Fig. 5.**
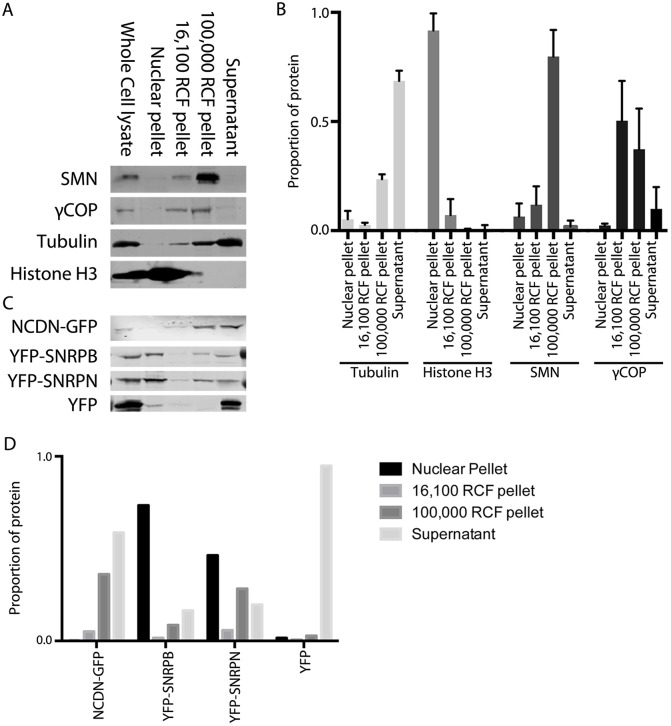
**Detergent-free fractionation of SH-SY5Y cells reveals that SMN, coatomer proteins, NCDN, SNRPB and SNRPN are all enriched in the 100,000 *g* vesicle pellet.** (A) Immunoblotting of equal protein amounts from fractionated SH-SY5Y cells reveals that SMN (top row) is highly enriched in the 100,000 ***g*** (RCF) pellet (small membrane-bound structures), with smaller amounts seen in the 16,100 ***g*** pellet (larger membrane-bound structures) and the nuclear pellet. The coatomer protein, γCOP (also known as COPG1; second row) is also enriched in the 100,000 ***g*** pellet as well as the 16,100 ***g*** pellet. Antibodies against histone H3 and tubulin confirm minimal nuclear contamination in cytoplasmic fractions, and minimal cytoplasmic contamination in the nuclear pellet, respectively. (B) Quantification of immunoblot analysis confirms that SMN is highly enriched in the 100,000 ***g*** pellet, with enrichment of γCOP also seen. Histone H3 and tubulin are highly enriched in the nucleus and cytoplasm, respectively. Quantification (mean±s.d.) of tubulin and histone H3 band density was from seven immunoblots, with values from SMN and γCOP from five and four immunoblots, respectively. (C) Immunoblotting of equal protein amounts from fractionated SH-SY5Y cells constitutively expressing NCDN–GFP, YFP–SNRPB, YFP–SNRPN or YFP alone (all detected with anti-GFP antibody) reveals that NCDN–GFP is enriched in the 100,000 ***g*** pellet, with smaller amounts seen in the 16,100 ***g*** pellet and the cytosolic supernatant. YFP–SNRPB and YFP–SNRPN are both also found in the 100,000 ***g*** pellet, in addition to the nuclear pellet and cytosolic supernatant. YFP alone is found almost exclusively in the cytosolic supernatant, with none detected in the 100,000 ***g*** or 16,100 ***g*** pellets. (D) Quantification of the band densities for the immunoblot shown in C confirms the presence of NCDN–GFP, YFP–SNRPB and YFP–SNRPN in the 100,000 ***g*** pellet, together with the restriction of YFP alone to the residual cytosolic supernatant.

To further investigate the possibility that the interaction of NCDN with SMN and the Sm proteins occurs within cytoplasmic vesicles, subcellular fractionations were performed on both parental SH-SY5Y cells and SH-SY5Y cell lines constitutively expressing NCDN–GFP, YFP–SNRPB, YFP–SNRPN or YFP. Sequential centrifugation was used to separate the cells into a nuclear fraction, 16,000 ***g*** and 100,000 ***g*** cytoplasmic pellets, and cytosolic supernatant (De Duve, 1971). Immunoblotting of these subcellular fractions revealed that GFP–NCDN, YFP–SNRPB and YFP–SNRPN, were all enriched in the 100,000 ***g*** cytoplasmic pellet, along with endogenous SMN and coatomer proteins (Fig. 5). This fraction would be expected to contain membrane-bound structures, such as microsomes and small cytoplasmic vesicles, which would encompass small coatomer-type endocytic vesicles. Endogenous SNRPN was also observed to enrich similarly (Fig. S3). A larger proportion of NCDN–GFP remained in the cytosolic supernatant than did YFP–SNRPB, YFP–SNRPN and endogenous SMN, which is in agreement with the subcellular localisations observed (Fig. 4). This further supports our hypothesis that the interactions between NCDN, SMN and the Sm proteins take place in small cytoplasmic vesicles.

### NCDN is required for the correct subcellular localisation of SMN

**Fig. 6.**
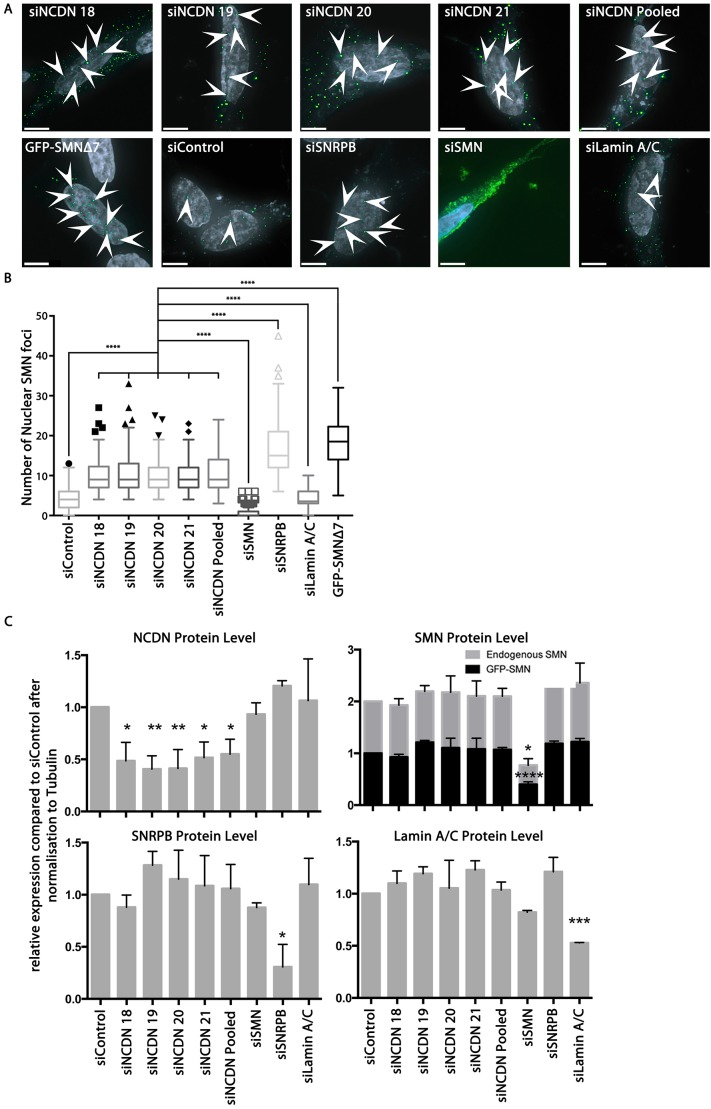
**Reduction of endogenous NCDN through siRNA increases localisation of SMN to nuclear foci.** (A) Transfection of SH-SY5Y cells constitutively expressing GFP–SMN (green) with siRNAs shows an increase in the number of SMN-positive nuclear foci (arrowheads) in cells transfected with siRNAs against NCDN (siNCDN, pooled represents all four siRNA molecules together) or SNRPB (siSNRPB), and a decrease in the number of SMN-positive nuclear foci in cells transfected with siRNAs against SMN (siSMN) in comparison to cells transfected with non-targeting siRNAs (siControl) or siRNAs against lamin A/C (siLamin A/C) as a ‘targeting’ control. Transfection of SH-SY5Y cells with a plasmid to express GFP–SMNΔ7 also results in increased numbers of SMN-positive nuclear foci. Cell nuclei are stained with Hoechst 33342 (grey on images). Transfection efficiency with siRNAs was greater than 90%, measured by transfection with siGlo Cyclophillin B (data not shown). Scale bars: 7 µm. Images are deconvolved *z*-stacks taken with 0.2 µm spacing. (B) Quantification of numbers of SMN-positive nuclear foci per nucleus showing that there is a significant increase following reduction of NCDN [10.2 (±4.1) with siNCDN 18, 10.4 (±5.0) with siNCDN 19, 9.9 (±4.1) with siNCDN 20, 9.5 (±3.6) with siNCDN 21, and 10.7 (±4.6) with siNCDN pooled] compared to 4.4 (±2.5) in cells treated with non-targeting siRNA (siControl) and 4.2 (±2.3) in cells treated with siRNAs targeting lamin A/C (siLaminA/C). Reduction of SNRPB also shows an increase in numbers of SMN-positive nuclear foci [to 16.7 (±6.8) with siSNRPB], while reduction of SMN leads to a decrease in numbers of SMN-positive nuclear foci [to 0.7 (±1.4) with siSMN]. Expression of GFP–SMNΔ7 results in an increase of numbers of SMN-positive nuclear foci to 18.2 (±5.3). All values are mean±s.d. The difference between each siNCDN and controls is statistically significant (AVOVA; *P*<0.0001, *n*=150 from 3 replicates). The box represents the 25–75th percentiles, and the median is indicated. The whiskers show the range of the data excluding outliers identified by the Tukey method. A Tukey post-test identified outliers (individual points marked on graph). (C) Immunoblot analysis using antibodies to endogenous NCDN, SMN and SNRPB shows a reduction in expression of each of 40–60% compared to siControl cells, after signals were normalised to tubulin (also see Fig. S4). Reductions in protein expression (mean±s.d.) compared to siControl siRNA are statistically significant (ANOVA; *P*<0.0001 for NCDN, Lamin A/C and endogenous SMN; *P*<0.001 for SNRPB; *P*<0.01 for GFP–SMN, *n*=3). A Dunnett post-test identified the significance of the reduction compared to siControl. *P≤0.05, **P≤0.01, ***P≤0.001, ****P≤0.0001.

We have previously documented that reduction of SNRPB expression results in re-localisation of SMN into numerous nuclear structures, probably analogous to gems (gemini of CBs), and its loss from cytoplasmic structures (Prescott et al., 2014). To investigate the requirement for NCDN in cytoplasmic SMN localisation, an SH-SY5Y cell line constitutively expressing GFP–SMN (Clelland et al., 2009) was transfected with siRNAs targeting NCDN [four different single siRNAs (Dharmacon) and a pooled sample]. A reduction in NCDN expression caused an increase in the number of SMN-positive nuclear foci present in the cell nucleus, as did a reduction of SNRPB expression (Fig. 6A,B). Conversely, reduction in SMN expression reduced the number of SMN-positive nuclear foci. The use of non-targeting control (siControl) sequences or positive control siRNAs (targeting lamin A/C) had no effect on the number of SMN-positive nuclear foci. The reduction in gene expression, as assayed by immunoblotting, for each siRNA was typically 40–60% (Fig. 6C). This suggests that NCDN is required for the correct sublocalisation of SMN. Of potential relevance for SMA pathology, depletion of either NCDN or SNRPB causes GFP–SMN to adopt a subcellular localisation reminiscent of that shown by GFP–SMNΔ7 (Fig. 6), a truncated version of SMN that mimics the product of the *SMN2* gene and is unable to completely substitute for full-length SMN in models of SMA (Le et al., 2005; Monani et al., 1999, 2000).

### SMN is required for the correct subcellular localisation of NCDN

**Fig. 7.**
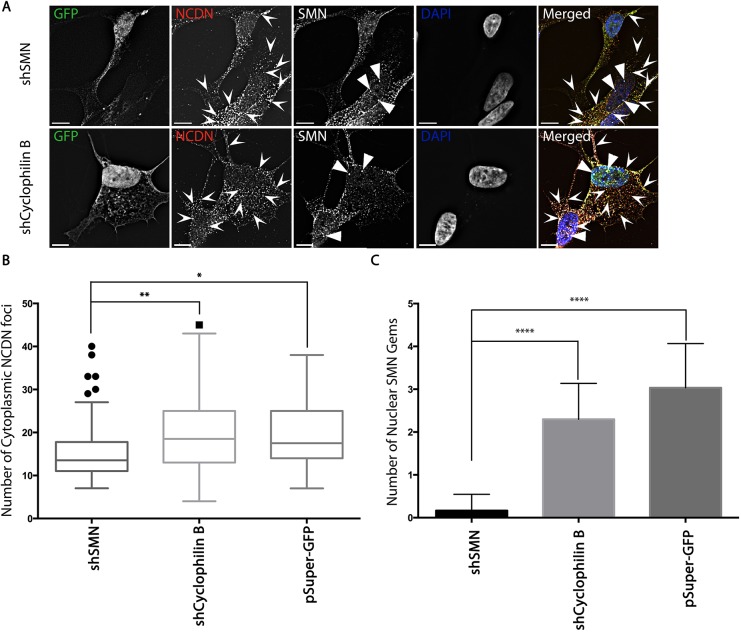
**Reduction of endogenous SMN causes a reduction in cytoplasmic NCDN foci in SH-SY5Y cells.** (A) SH-SY5Y cells were transfected with plasmids to express shRNAs targeting SMN (shSMN), Cyclophilin B (shCyclophilin) or with the empty pSuper GFP vector (data not shown), fixed after 72 h, and immunostained for endogenous NCDN and SMN allowing detection of NCDN foci within the cytoplasm (chevron arrowheads), as well as SMN-positive nuclear gems (triangular arrowheads). Images are single deconvolved z-sections. Scales bars: 7 µm. (B) The depletion of SMN results in a reduction in the number of NCDN foci present in the cytoplasm to 15.3 (±7.2) (mean±s.d.) from 20.6 (±12.0) and 19.5 (±7.6) compared to cells transfected with either shCyclophilin B or the empty pSuper GFP vector, respectively (ANOVA, *P*<0.0005, *n*=64 from 3 replicates). The box represents the 25–75th percentiles, and the median is indicated. The whiskers show the range of the data excluding outliers identified by the Tukey method. Outliers are shown as individual points. (C) The depletion of SMN causes a reduction of nuclear gems to 0.17 (±0.38) from 2.3 (±0.84) or 3.0 (±1.0) compared to cells transfected with shCyclophilin B or empty vector respectively confirming efficient reduction in SMN protein levels. Results are mean±s.d. (*n*=30 from three replicates). *P≤0.05, **P≤0.01, ***P≤0.001, ****P≤0.0001 (ANOVA).

To investigate whether NCDN requires the SMN protein for its localisation to vesicles in neural cells, SH-SY5Y cells were transfected with shRNA constructs previously validated to reduce the expression of SMN by an average of 46%, a reduction previously found to cause symptoms resembling SMA type III in mouse models (Jablonka et al., 2000), and carrying a GFP marker to unequivocally identify transfected cells (Clelland et al., 2012). Reduction of SMN, monitored by quantification of the number of SMN-positive nuclear foci (Fig. 7A,C), reduced the number of cytoplasmic foci containing endogenous NCDN (Fig. 7A,B). This, together with data in Fig. 6, suggests that NCDN and SMN are mutually dependent for their incorporation into cytoplasmic structures, raising the possibility that the lowered levels of SMN seen in SMA could compromise NCDN function.

### NCDN does not co-purify with splicing snRNPs, suggesting it is not involved in snRNP assembly

**Fig. 8.**
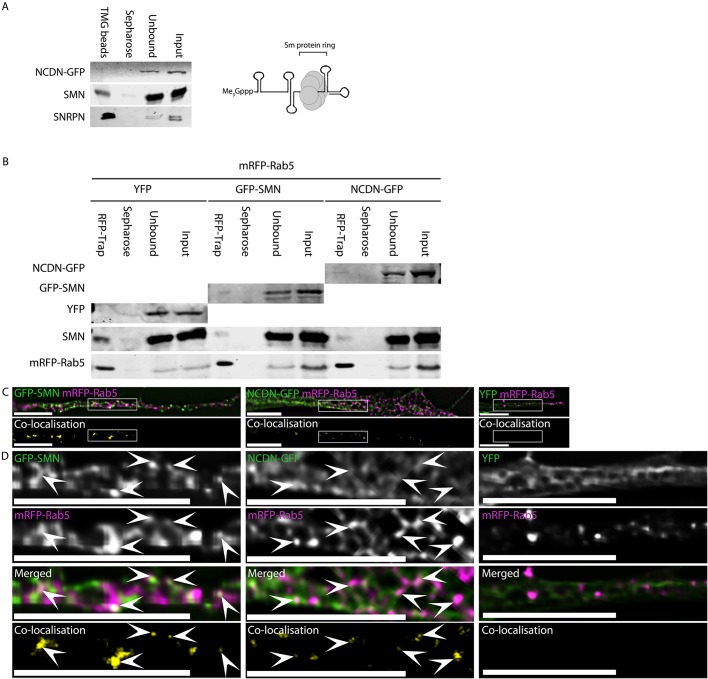
**NCDN does not co-purify with snRNPs, while NCDN and SMN interact with Rab5 and colocalise with a subset of Rab5 vesicles within neurites of SH-SY5Y cells.** (A) Incubation of whole-cell lysate from an SH-SY5Y cell line constitutively expressing NCDN–GFP with agarose beads conjugated to antibodies against the tri-methyl guanosine cap (Me3Gppp) of snRNAs (TMG beads) affinity purifies snRNPs as evidenced by the enrichment of the core snRNP protein SNRPN (detected with anti-SNRPN antibody, bottom row). The enriched snRNP fraction also contains SMN, which is essential for snRNP assembly. NCDN–GFP, however, does not co-enrich with snRNPs. Also shown is the core structure of mature snRNPs consisting of the heptameric Sm protein ring bound at the Sm-binding site of snRNA, as well as the characteristic tri-methyl guanosine Cap of snRNAs (Me_3_Gppp) at the 5′ end. (B) Affinity isolation of mRFP–Rab5 using RFP-Trap from cells co-transfected with plasmids to express mRFP–Rab5 together with NCDN–GFP, GFP–SMN or YFP alone co-enriches both NCDN–GFP (top row, detected with anti-GFP antibody, band is present in RFP-Trap lane but not Sepharose beads lane) and SMN-GFP (second row, detected with anti-GFP antibody, band is present in RFP-Trap lane but not Sepharose beads lane), but not YFP (third row, no band detected in RFP-Trap lane). Endogenous SMN (fourth row, detected with mouse anti-SMN) co-enriches with mRFP–Rab5 in all three samples. Detection of mRFP–Rab5 (bottom row, detected with anti-RFP antibody) confirms substantial enrichment of mRFP–Rab5 in all three samples. (C) Both GFP–SMN and NCDN–GFP partially colocalise with mRFP–Rab5 in a subset of mRFP–Rab5-containing vesicles in co-transfected SH-SY5Y cells (white signal in overlaid images, top row; yellow signal in colocalisation images, bottom row). (D) Enlargement of the boxed areas in C confirms that the colocalisation between SMN or NCDN and Rab5 occurs in punctate structures. Arrowheads identify areas of colocalisation. Colocalisation images were generated by Volocity using automatic thresholds on non-deconvolved *z*-sections before excluding values below 0.05. Images (excluding the colocalisation images) are single deconvolved *z*-sections. Scale bars: 7 µm.

To investigate whether the interaction between NCDN and SMN could reflect a previously unidentified role for NCDN in snRNP assembly, splicing snRNPs were affinity purified from whole-cell lysates of SH-SY5Y cells constitutively expressing NCDN–GFP using agarose beads coupled to antibodies against the characteristic tri-methyl guanosine Cap of snRNAs (TMG beads, Millipore) (Fig. 8A). Endogenous SNRPN protein showed strong enrichment in the affinity purified snRNP samples, as expected for a core snRNP protein. Endogenous SMN was also co-enriched with snRNPs, demonstrating that the experimental conditions were suitable to identify proteins important for snRNP assembly as well as those that are genuine snRNP components. NCDN–GFP did not co-purify with splicing snRNPs, however, suggesting that NCDN is not involved in snRNP assembly or processing. This raises the intriguing possibility that the interaction between SMN and NCDN reflects a novel, snRNP-independent role for SMN.

### SMN interacts with Rab5 in SH-SY5Y cells and colocalises with a sub-set of Rab5 vesicles

Recent studies have found that SMA may cause endocytic defects, especially in synaptic vesicle recycling in animal models (Dimitriadi et al., 2016). Several SMA-protective disease modifier genes, such as plastin 3, coronin 1C and neurocalcin δ, are also associated with endocytosis (Hosseinibarkooie et al., 2016; Oprea et al., 2008; Riessland et al., 2017). However, other endocytic structures within the cell have not been investigated. Rab5 (which has two forms, Rab5a and Rab5b) is a marker of early endosomes and endocytic vesicles, as well as being a regulator of these trafficking pathways (Bucci et al., 1992). NCDN and Rab5 have previously been shown to interact, while both Rab5 and NCDN both have roles in dendrite morphogenesis and cell polarity (Guo et al., 2016; Oku et al., 2013; Satoh et al., 2008).

As we have shown above that SMN and NCDN colocalise in vesicles, we hypothesised that some of the SMN-rich vesicles could be Rab5 vesicles. SH-SY5Y cells were co-transfected with plasmids to express mRFP–Rab5 (the Rab5a form) (Vonderheit and Helenius, 2005) together with either GFP–SMN, NCDN–GFP or YFP. mRFP–Rab5 was affinity-purified from whole-cell lysates from each co-transfection by using RFP-TRAP (Chromotek). Subsequent immunoblotting revealed co-purification of GFP–SMN and NCDN–GFP, but not YFP alone, with mRFP–Rab5 (Fig. 8B). Furthermore, endogenous SMN also co-purified with mRFP–Rab5. In parallel experiments, colocalisation of mRFP–Rab5 with GFP–SMN and NCDN was investigated (Fig. 8C,D). In accordance with previous publications, Rab5 showed partial colocalisation with NCDN–GFP in cytoplasmic structures (arrows in Fig. 8D) (Oku et al., 2013). GFP–SMN showed a similar degree of colocalisation with mRFP–Rab5, also in cytoplasmic structures, while there was minimal colocalisation between YFP and mRFP–Rab5. Taken together with the absence of NCDN from enriched snRNP fractions (Fig. 8A), this suggests that NCDN and SMN colocalise in the context of Rab5 vesicles, independently of snRNP assembly.

## DISCUSSION

The genetic cause of SMA has been known since 1995 (Lefebvre et al., 1995), but there is still little available in the way of treatment. Spinraza/Nusinersen is now available to treat SMA by correcting the defective splicing of the *SMN2* transcript to promote production of full-length SMN protein. However, this is not a complete cure and requires regular maintenance doses through intrathecal injection. Additionally, little has been done to investigate potential symptoms that could arise later in life or in other tissues and organs in patients treated with Spinraza. Additional treatment options for SMA are still needed for use in addition to Spinraza, or in place of it for patients for whom it is not suitable, including those with rarer forms of SMA in which SMN1 is not mutated.

A significant reason for the lack of treatment options for SMA is uncertainty about the cellular roles of SMN, which appear to be numerous. In particular, it is not clear why motor neurons are so exquisitely sensitive to reduced levels of SMN when the key roles of the protein appear to be in pathways required in all cell types. By comparing the interactomes of two very similar members of Sm protein family, SNRPB and the neural-specific SNRPN, we have uncovered an interaction between SMN and the essential neural protein NCDN, which may be of relevance for SMA pathology and have the potential to open novel avenues for therapy development.

### The neural-specific Sm protein SNRPN behaves similarly to SNRPB, but shows subtle differences at the interactome level that may indicate alternative roles

Differences between members of the Sm protein family have not been systematically investigated, although non-splicing roles have been proposed for SNRPB and SNRPD3 in mRNA localisation, and for SNRPD1 in miRNA biogenesis, in *Drosophila* (Gonsalvez et al., 2010; Xiong et al., 2015). As SNRPB and SNRPN are thought to perform the same primary function in snRNPs (Huntriss et al., 1993), it is currently unknown why SNRPN is expressed in neural tissues as well as, or instead of, SNRPB. Current research has suggested that the expression of SNRPN may cause tissue-specific alternative splicing of pre-mRNA transcripts (Lee et al., 2014). However, an alternative, but complementary hypothesis is that SNRPN may be adapted for secondary neural-specific roles. We demonstrate here that SNRPN localises identically to SNRPB during snRNP maturation and at the steady-state, when both localise to vesicles containing SMN in the cytoplasm and neurites of SH-SY5Y cells in addition to their canonical localisation to nuclear CBs and speckles. Our parallel proteomic study used SH-SY5Y neural cell lines constitutively expressing YFP–SNRPN and YFP–SNRPB to investigate differences between the interactomes of these two, very similar, proteins. SNRPN has a proline-rich C-terminal tail that SNRPB lacks, although a similar sequence is present in SNRPB′ (Mourao et al., 2016), an alternatively spliced product of the *SNRPB* gene. Several proteins were identified in the SNRPN interactome but not the SNRPB interactome, such as nuclear receptor co-activator 6 interacting protein (UniProt Q96RS0), and 7SK snRNA methylphosphate capping enzyme (UniProt Q7L2J0), both of which are associated with snRNA capping (Hausmann et al., 2008; Jeronimo et al., 2007). Additionally, RNA-binding protein 40 (UniProt Q96LT9) was uniquely identified within the SNRPN interactome, and is involved in the minor spliceosome (Benecke et al., 2005). This suggests that some of these proteins may interact preferentially with SNRPN, perhaps mediated by amino acid changes within the proline-rich tail. However, further validation and additional experimentation would be required to confirm these differences in interactome between SNRPN and SNRPB, and to investigate specific functions for the distinct Sm protein family members.

### NCDN interacts with SMN, SNRPB and SNRPN and colocalises with them in vesicles, suggesting a novel cellular role for SMN

Previous research into neural-specific functions for SMN has identified several new protein–protein interactions involving SMN. These novel SMN partners have, in the main, been RNA-binding proteins (Akten et al., 2011; Fallini et al., 2014, 2011; Rossoll et al., 2003). There is growing appreciation that SMN-mediated transport may be of particular importance in neural cells and involve COPI-type vesicles transported by dynein and containing SNRPB (Custer et al., 2013; Li et al., 2015; Peter et al., 2011; Prescott et al., 2014). The nature and content of these vesicles is not clear but they are likely to be of significance for the cell-type bias of SMA symptoms, as they are present predominantly in neural cells (Akten et al., 2011; Fallini et al., 2016, 2014, 2011; Li et al., 2015; Peter et al., 2011; Prescott et al., 2014; Rossoll et al., 2003; Todd et al., 2010a,b; Zhang et al., 2006, 2003). The SNRPN/SNRPB interactome screen presented here suggests a large number of non-snRNP proteins as potential cellular partners for the Sm proteins.

We chose to investigate the neural protein NCDN further, as it has characteristics that may be of relevance for SMA. NCDN is predominantly expressed in neural tissue, and little is known about its structure or function, as it shares little sequence similarity to other eukaryotic proteins (Shinozaki et al., 1997). Although characterised relatively poorly, it is associated with dendrite morphogenesis and localises to Rab5 vesicles, which are involved in the maintenance of cell polarity (Guo et al., 2016; Oku et al., 2013). NCDN has also been demonstrated to regulate localisation of signalling proteins such as P-Rex 1 (Pan et al., 2016), suggesting that it, in common with SMN, has a role in intracellular trafficking. These neural-specific and trafficking roles suggest that further analysis of the interaction between NCDN and the Sm proteins might lead to better understanding of the molecular mechanisms of pathogenesis in SMA.

Reciprocal affinity-purification of GFP-tagged and endogenous NCDN and Sm proteins (Fig. 3A,B) validated the interaction detected in the interactome analysis. Although originally identified as a protein interacting with SNRPN but not SNRPB, further investigation indicates that NCDN is, in fact, capable of interacting with both of these Sm proteins. Of much greater interest, however, is the interaction documented between NCDN and SMN, which appears more robust than that between NCDN and the Sm proteins (Fig. 3). Furthermore, NCDN localises with SMN and the Sm proteins in mobile vesicles in the neurites of SH-SY5Y cells (Fig. 4), rather than in the nucleus, suggesting that it shares cytoplasmic, rather than nuclear, roles with SMN. The truncated protein SMNΔ7, which cannot fully substitute for full-length SMN despite retaining some functionality, is largely restricted to the nucleus (Renvoise et al., 2006; Sleigh et al., 2011). While it cannot yet be ruled out that SMNΔ7 lacks the capability to substitute for full-length SMN in nuclear roles, this suggests that cytoplasmic roles of SMN are key to SMA pathology. NCDN was not co-purified with splicing snRNPs, under conditions that showed a clear enrichment of SMN, a key assembly factor for snRNPs, in the snRNP fraction. This suggests that the interaction between NCDN, SMN and the Sm proteins is not related to snRNP assembly.

### Potential consequences of NCDN mis-localisation associated with SMN reduction

We have identified colocalisation of both SMN and NCDN with a subset of Rab5 vesicles. Since NCDN is also found in a subset of SMN-positive cytoplasmic structures, it is highly likely that these are Rab5 vesicles. It is possible that the protein–protein interactions between NCDN and SMN occur elsewhere in the cytoplasm as both proteins also show a diffuse cytosolic pool, but the decrease seen in cytoplasmic structures containing NCDN following SMN depletion suggests that cellular pathways requiring NCDN-containing vesicles may be compromised in SMA. Loss of NCDN-positive cytoplasmic structures was seen in cells with a moderate reduction in SMN levels, so NCDN may be of relevance for patients with milder forms of SMA.

At present, the precise roles of NCDN are not fully understood, although it has been implicated in dendrite morphogenesis, neural outgrowth, synaptic plasticity regulation and moderation of signalling pathways in neural cells (Dateki et al., 2005; Francke et al., 2006; Matosin et al., 2015; Ohoka et al., 2001; Oku et al., 2013; Pan et al., 2016; Shinozaki et al., 1999, 1997; Wang et al., 2013, 2009; Ward et al., 2009). NCDN has also previously been shown to localise to Rab5 vesicles within dendrites (Oku et al., 2013). These dendritic Rab5 vesicles have been found to have an important role in dendrite morphogenesis and somatodendritic polarity (Guo et al., 2016; Satoh et al., 2008). As we have now demonstrated that SMN localises to a subset of Rab5 vesicles, likely in association with NCDN, SMN may also be implicated in cell polarity, with an insufficiency of SMN causing problems with both establishment and maintenance of polarity. These would be particularly vital in such elongated cells as motor neurons and may be mediated through trafficking of mRNAs or proteins. Further work will be required to investigate defects in cell polarity as a pathogenic mechanism in SMA and their possible link to NCDN.

### NCDN as a potential novel therapeutic target in SMA

SMN has now been linked to several functions other than its canonical role in snRNP assembly. While reduction in the capacity of the cell for snRNP assembly caused by lowered SMN may cause splicing defects, the key transcripts preferentially affecting motor neurons are still to be identified. SMN has an established role in the trafficking of mature mRNAs destined for localised translation. The nature of the structures involved in this role is not completely clear, however, with different authors describing the structures as vesicular or granular. Reduction of SMN has also been linked with endosomal defects, suggestive of the importance of SMN for vesicular transport. Here, we provide further evidence for the presence of SMN in, or associated with, vesicles and document interactions between the essential neural protein, NCDN and SMN. Together with the clear enrichment of NCDN in motor neurons in mouse spinal cord, this suggests that NCDN may be a downstream target of SMN reduction in SMA, and places it as a potential target for therapy development in SMA. Further work will be required to establish which roles of NCDN also involve SMN, and whether these are of relevance for the molecular pathology of SMA. The co-dependence of SMN and NCDN in cytoplasmic vesicles, however, suggests that depletion of SMN, as seen in the majority of SMA patients, may affect NCDN localisation and/or function.

## MATERIALS AND METHODS

### Plasmid constructs

pEGFP-SMN, pEYFP-SNRPB and mCherry-SNRPB have been described previously (Clelland et al., 2009; Sleeman et al., 2001; Sleeman and Lamond, 1999). pEYFP-SNRPN and pmCherry-SNRPN were generated by PCR amplification and subcloning cDNA of human SNRPN from SH-SY5Y cells into pEYFP-C1 and pmCherry-C1, respectively, by using SNRPNEcoRI forward primer 5′-TAGAATTCCATGACTGTTGGCAAGAGTAGC-3′, and SNRPNBamHI reverse primer 5′-TAGGATCCCTGAGATGGATCAACAGTATG-3′. pmCherry-SMN was generated by PCR amplification and subcloning the sequence from the pEGFP-SMN plasmid into pmCherry-C1 using an SMNEcoRI forward primer 5′-GCGGAATTCTATGGCGATGAGC-3′ and SMNBamHI reverse primer 5′-GCAGGATCCTTAATTTAAGGAATGTGA-3′. To generate pEGFP-NCDN, NCDN cDNA from SH-SY5Y cells was PCR amplified and subcloned into a pEGFP-N3 plasmid using NCDNEcoRI forward primer 5′-GCGGAATTCATGGCCTCGGATTGCG-3′ and NCDNSalI reverse primer 5′-GCTGCTGACGGGCTCTGACAGGC-3′. All cDNAs were amplified using GoTaq G2 (Promega, Madison, WI) and the PCR products restriction digested using EcoRI and either BamHI or SalI (Promega), before ligation with T4 DNA ligase (Thermo Scientific, Waltham, MA). mRFP-Rab5 was a gift from Ari Helenius (Institut für Biochemie, ETH Zurich, Zürich, Switzerland) (Vonderheit and Helenius, 2005).

### Cell lines and cell culture

SH-SY5Y cells were from the ATCC. Cells were cultured in DMEM with 10% FBS at 37°C, 5% CO_2_. Transfections were carried out using Effectene (Qiagen, Hilden, Germany) according to the manufacturer's instructions. Stable SH-SY5Y cell lines expressing mCherry–SNRPB and GFP–SMN have been described previously (Clelland et al., 2009; Prescott et al., 2014). SH-SY5Y cell lines stably expressing YFP–SNRPN, YFP–SNRPB, YFP, mCherry–SNRPN and NCDN–GFP were derived by clonal isolation following selection with 200 µg/ml G418 (Roche, Basel, Switzerland) following transfection.

### Animals

Mouse tissues in this study were obtained from littermate healthy control mice (*Smn^+/−^; SMN2^tg/0^*) from the ‘Taiwanese’ model of SMA (Hsieh-Li et al., 2000). Mice were originally obtained from Jackson Laboratories and were maintained in animal care facilities at the University of Edinburgh, UK under standard specific pathogen-free conditions on a congenic FVB background. All animal breeding was performed in accordance with University of Edinburgh institutional guidelines and under the appropriate project and personal licences granted by the UK Home Office.

### Immunostaining, microscopy and image analysis

Cell fixing and immunostaining were both carried out as described previously (Sleeman et al., 2003). Immunostaining of spinal cord sections was carried out as described previously (Powis and Gillingwater, 2016). Live-cell and fixed cell microscopy and image processing were carried out as described previously (Prescott et al., 2014). BODIPY-493 (Life Technologies, Paisley, UK) was added to culture medium at 2 µg/ml overnight. Antibodies used for immunostaining were: mouse monoclonal Y12 anti-Smith (SNRPB) (Abcam, Cambridge, MA, ab3138, 1:20), rabbit polyclonal 204-10 (anti-Coilin) (a gift from Angus I. Lamond, School of Life Sciences, University of Dundee, UK; 1:500; Bohmann et al., 1995), mouse monoclonal anti-SMN (BD Transduction, San Jose, CA, 610646, 1:50), goat anti-ChAT (Millipore, Burlington, MA, AB144P) and rabbit polyclonal anti-NCDN (Proteintech, Manchester, UK, 13187-1-AP, 1:50) antibodies. Overlays of images were made using Adobe Photoshop CS5 (Adobe, San Jose, CA). Colocalisation images were generated by using Volocity 6.3 (PerkinElmer, Waltham, MA) with automatic thresholds on non-deconvolved images. Colocalisation values of 0.05 or less were excluded as this was the maximum colocalisation value observed between the mCherry signal and YFP signal in neurites expressing YFP as a control together with mCherry-tagged proteins of interest. Deconvolution was also performed using Volocity 6.3, with between 15 and 25 iterations of deconvolution. Images of spinal cord sections collected using a 60× objective on a DeltaVision RT microscope (Applied Precision) were assembled into panels using the FIJI (Schindelin et al., 2012) plug-in and a custom written export protocol.

### Preparation of cell lysates and immunoblotting

Cells were grown in 10 cm diameter dishes, before being detached with trypsin and collected by centrifugation at 180 ***g*** for 5 min. The cell pellet was washed three times in PBS before lysis in 100 µl of ice-cold lysis buffer per dish [50 mM Tris-HCl pH 7.5, 0.5 M NaCl; 1% (v/v) Nonidet P-40, 1% (w/v) sodium deoxycholate, 0.1% (w/v) SDS, 2 mM EDTA plus cOmplete mini EDTA-free protease inhibitor cocktail (Roche, one tablet per 10 ml)], followed by homogenisation by sonication. Isolation of YFP/GFP and mCherry/mRFP-tagged proteins was carried out as described previously with GFP- or RFP-Trap (Chromotek, Planegg-Martinsried, Germany) (Prescott et al., 2014). Immunoprecipitation of endogenous SMN from brain lysate was carried out as described previously by using mouse monoclonal anti-SMN (BD Transduction Labs 610646) (Boyd et al., 2017; Groen et al., 2013). Lysates were electrophoresed on a 10% SDS-polyacrylamide gel and transferred onto nitrocellulose (Hybond-C+ or Protran premium 0.2 µm, both GE Healthcare, Little Chalfont, UK) membranes for immunoblotting. Antibodies used were rat monoclonal anti-RFP (Chromotek 5F8, 1:500); goat polyclonal anti-γCOP (Santa Cruz Biotechnology, Dallas, TX, sc-14167, 1:250), rabbit polyclonal anti-GFP (Abcam ab290, 1:2000), rabbit polyclonal anti-SNRPN (Proteintech 11070-1-AP, 1:800), mouse monoclonal Y12 anti-Smith (SNRPB) (Abcam ab3138, 1:100), rabbit polyclonal anti-SMN (Santa Cruz Biotechnology, sc-15320, 1:500), mouse monoclonal anti-SMN (BD Transduction Labs 610646, 1:500), rabbit polyclonal anti-COPB1 (CUSAB, College Park, MD, CSB-PA005783LA01HU, 1:500), mouse monoclonal anti-Lamin A/C (Santa Cruz Biotechnology, sc-7292, 1:500), rabbit polyclonal anti-NCDN (Proteintech 13187-1-AP, 1:500), mouse monoclonal anti-tubulin (Sigma Aldrich, St Louis, MO, 1:500) and rabbit polyclonal anti-Histone H3 (Proteintech 17168-1-AP, 1:300). Secondary antibodies were goat anti-rabbit-IgG conjugated to Dylight 700 (Thermo Scientific 35569, 1:15,000) or goat anti-mouse-IgG conjugated to Dylight 800 (Thermo Scientific SA5-10176, 1:15,000). Alternatively, goat anti-mouse-IgG conjugated to IRDye 800CW (Li-Cor 925-32210, 1:25,000) and goat anti-rabbit-IgG conjugated to IRDye 680RD (Li-Cor 925-68071, 1:25,000) were used. Goat anti-rat-IgG conjugated to Dylight 800 (Thermo Scientific, SA5-10024) antibody was used to visualise the rat monoclonal anti-RFP antibody at a concentration of 1:15,000. Donkey anti-goat-IgG conjugated to IRDye 800CW (Licor, Lincoln, NE, 925-32214) was used at a concentration of 1:25,000 to detect goat polyclonal anti-γCOP antibody. Donkey anti-rabbit-IgG conjugated to horseradish peroxidase (HRP) (Pierce, Waltham, MA, 31460, 1:15,000) was used to identify endogenous NCDN in Fig. 3D. Detection of antibodies conjugated to fluorophores was carried out with an Odessey CLx using Image Studio (both Li-cor). Band quantification was also performed by using Image Studio software. Detection of antibodies conjugated to HRP was performed using ECL Western Blotting Substrate (Pierce) and developed with Hyperfilm (Amersham), using a Kodak X-OMAT 1000 developer, after 30-45 min exposure.

### Immunoprecipitation of intact snRNPs

To immunoprecipitate intact snRNPS, whole-cell lysates were incubated with anti-2,2,7-trimethylguanosine conjugated to agarose beads (Millipore NA02A), with Sepharose 4B beads (Sigma-Aldrich) as a control. 40 ng of pre-cleared lysate and unbound protein were separated by SDS-PAGE alongside material precipitated with Sepharose control beads and TMG antibody beads. Subsequent detection was carried out using rabbit anti-GFP (1:2000, Abcam), rat mAb anti-RFP (1:500, Chromotek), mouse monoclonal anti-SMN (1:500, BD Transduction Labs) and rabbit polyclonal anti-SNRPN (1:800, Proteintech) antibodies.

### Preparations and analysis of MS samples

SH-SY5Y cells constitutively expressing YFP, YFP–SNRPN or YFP–SNRPB were lysed in co-immunoprecipitation buffer [10 mM Tris-HCl pH 7.5, 150 mM NaCl, 0.5 mM EDTA, 0.5% NP40, 1 cOmplete EDTA-free protease inhibitor tablet (Roche) per 10 ml], followed by affinity purification of the tagged proteins with GFP-Trap as above. 11 mg of total protein per sample was used as input. 5 µl of the affinity isolated material, alongside precleared lysate and unbound lysate was transferred to nitrocellulose membrane (as above) and immunodetected using rabbit anti-GFP antibody (Abcam) to confirm efficient immunoprecipitation. Samples were then electrophoresed on a NuPAGE 4-12% Bis-Tris Acrylamide gel (Novex, Waltham, MA, NP0321), Coomassie stained using SimplyBlue SafeStain (Invitrogen, Paisley, UK), and gel chunks excised and analysed by the Mass Spectrometry and Proteomics Facility at the University of St Andrews.

The gel chunks were cut into 1 mm cubes. These were then subjected to in-gel digestion, using a ProGest Investigator in-gel digestion robot (Digilab, Hopkinton, MA) using standard protocols (Shevchenko et al., 1996). Briefly, the gel cubes were destained by washing with MeCN and subjected to reduction with DTT and alkylation with IAA before digestion overnight with trypsin at 37°C. The peptides were extracted with 10% formic acid, and the volume reduced to ∼20 µl by concentration in a speedvac (Thermo Scientific).

The peptides were then injected onto an Acclaim PepMap 100 C18 trap and an Acclaim PepMap RSLC C18 column (Thermo Scientific), using a nanoLC Ultra 2D plus loading pump and nanoLC as-2 autosampler (Eksigent). The peptides were eluted with a gradient of increasing acetonitrile, containing 0.1% formic acid (2–20% acetonitrile in 90 min, 20–40% in a further 30 min, followed by 98% acetonitrile to clean the column, before re-equilibration to 2% acetonitrile). The eluate was sprayed directly into a TripleTOF 5600 electrospray tandem mass spectrometer (Sciex, Foster City, CA) and analysed in Information Dependent Acquisition (IDA) mode, performing 250 ms of MS followed by 100 ms MS/MS analyses on the 20 most intense peaks seen by MS. The MS/MS data files generated were analysed using the ProteinPilot Paragon search algorithm v5.0.1 (Sciex) against the NCBInr database (Nov 2012) restricting the search to *Homo sapiens*, with trypsin as the digestion enzyme and selecting cysteine alkylation with iodoacetamide, ‘Gel based ID’ as a ‘Special factor’, ‘Biological modifications’ as the ‘ID Focus’ and a ‘Thorough’ ‘Search effort’.

ProteinPilot also performs a decoy database search to assess the false discovery rate (FDR). Protein identifications were accepted if they were identified by at least two peptides with the Detected Protein Threshold set at 0.05. The data was also analysed via the ‘Create mgf file’ script in PeakView (Sciex) using the Mascot search algorithm (Matrix Science), against the NCBInr database (Oct 2014) restricting the search to *Homo sapiens* (284,317 sequences), trypsin as the cleavage enzyme and carbamidomethyl as a fixed modification of cysteine residues and methionine oxidation as a variable modification. The peptide mass tolerance was set to ±0.05 Da and the MS/MS mass tolerance to ±0.1 Da. Scaffold viewer (version Scaffold_4.5.1, Proteome Software) was used to validate the identifications from Mascot. Peptide identifications were accepted if they could be established at greater than 95.0% probability by the Peptide Prophet algorithm (Keller et al., 2002). Protein identifications were accepted if they could be established at greater than 99.0% probability and contained at least two identified peptides. Protein probabilities were assigned by the Protein Prophet algorithm (Nesvizhskii et al., 2003). Proteins that contained similar peptides and could not be differentiated based on MS/MS analysis alone were grouped to satisfy the principles of parsimony.

Identified proteins affinity purified alongside YFP–SNRPB or YFP–SNRPN were discounted if they were additionally identified as being affinity purified with YFP, or if they were present within the Sepharose bead proteome (Trinkle-Mulcahy et al., 2008). The MS proteomics data have been deposited to the ProteomeXchange Consortium via the PRIDE (Vizcaíno et al., 2016) partner repository with the dataset identifier PXD008710.

### RNAi assays

Reduction of protein expression using siRNA was achieved by transfecting the appropriate cell lines with siRNAs (Dharmacon, Lafayette, CO) using Viromer Green [Lipocalyx GmbH, Halle (Saale), Germany] according to the manufacturer's instructions. Cells were lysed for immunoblotting assays or fixed with paraformaldehyde for fluorescence microscopy at 48 h after transfection. siRNA sequences used were SMN: 5′-CAGUGGAAAGUUGGGGACA-3′; SNRPB, a mixture of 5′-CCCACAAGGAAGAGGUACU-3′, GCAUAUUGAUUACAGGAUG-3′, 5′-CCGUAAGGCUGUACAUAGU-3′ and 5′-CAAUGACAGUAGAGGGACC-3′; NCDN, individually and a mixture of NCDN 18, 5′-GUUCAUUGGUGACGAGAAA-3′, NCDN 19, 5′-AGACCUCAUCCUUGCGUAA-3′, NCDN 20, 5′-AGGCCAAGAAUGACAGCGA-3′ and NCDN 21, 5′-GGCCAUUGAUAUCGCAGUU-3′; negative control (siControl) targeting luciferase, 5′-UAAGGCUAUGAAGAGAUAC-3′; positive control targeting Lamin A/C, 5′-GGUGGUGACGAUCUGGGCU-3′; and SiGlo Cyclophillin B to determine transfection efficiency, 5′-GGAAAGACUGUUCCAAAAA-3′. Lysates were electrophoresed on an SDS-PAGE gel, transferred onto nitrocellulose membrane, and immunodetected with antibodies to the above proteins. Band signal intensity was determined with ImageStudio (Li-Cor), and the values were normalised to tubulin, following correction for background. Reduction of protein expression using shRNA was achieved by transfecting SH-SH5Y cell lines with pSUPER-GFP.Neo plasmids (Oligoengine, Seattle, WA) expressing shRNA to SMN and Cyclophilin B, which have been described previously (Clelland et al., 2012) using Effectene (QIAGEN).

### Fractionation

Cells were pelleted from the appropriate cell line, and incubated in Buffer A (10 mM HEPES pH 7.9, 1.5 mM MgCl_2_, 10 mM KCl, 0.5 mM DTT, 1 cOmplete EDTA-free protease inhibitor tablet per 10 ml) for 5 min, before being Dounce homogenised 25 times using the tight pestle to disrupt the plasma membrane. This was then centrifuged at 300 ***g*** for 5 min to pellet the nuclei. The supernatant was removed, recentrifuged at 300 ***g*** to further remove nuclei, before the supernatant was centrifuged at 16,100 ***g*** for 30 min using a refrigerated 5415R Centrifuge (Eppendorf, Hamburg, Germany). The nuclei were resuspended in buffer S1 (250 mM sucrose, 10 mM MgCl_2_), before this was layered over with buffer S3 (880 mM Sucrose, 0.5 mM MgCl_2_). The nuclear pellet was then centrifuged at 2800 ***g*** for 10 min to wash and pellet the nuclei. The supernatant from the 16,100 ***g*** centrifugation was further centrifuged at 100,000 ***g*** using an Optima Max-XP ultracentrifuge with a TLA-110 Rotor (Beckman-Coulter, Brea, CA) for 60 min. The supernatant was removed and kept. The 16,100 and 100,000 ***g*** pellets were washed in Buffer A and centrifuged at 16,100 ***g*** for 30 min or 100,000 RCF for 1 h, respectively. Each pellet was then resuspended in lysis buffer (see above). To confirm efficient separation of cytoplasmic fractions from the nuclear fractions, mouse anti-tubulin (Sigma-Aldrich, 1:500) and rabbit polyclonal anti-histone H3 (Proteintech, 1:300) antibodies were used.

### Statistical analysis and generation of graphs

Data was processed using Microsoft Excel to produce ratios, proportions and percentages. Bar charts and box-and-whisker plots were then generated by using Prism 6 (GraphPad, La Jolla, CA) from the processed data. Statistical analysis was also performed in Prism 6, with multiple comparisons to determine statistical difference between specific sets of data. Tukey post-tests were used to identify outliers in ANOVA statistical analysis.

## Supplementary Material

Supplementary information
