# Supplementary material for: Neurochondrin interacts with the SMN protein suggesting a novel mechanism for spinal muscular atrophy pathology
Source: J Cell Sci. 2018 Apr 17;131(8):jcs211482. doi: 10.1242/jcs.211482 (PMC5963842; doi:10.1242/jcs.211482)
Supplement: Supplementary information [file joces-131-211482-s1.pdf]

J. Cell Sci. 131: doi:10.1242/jcs.211482: Supplementary information

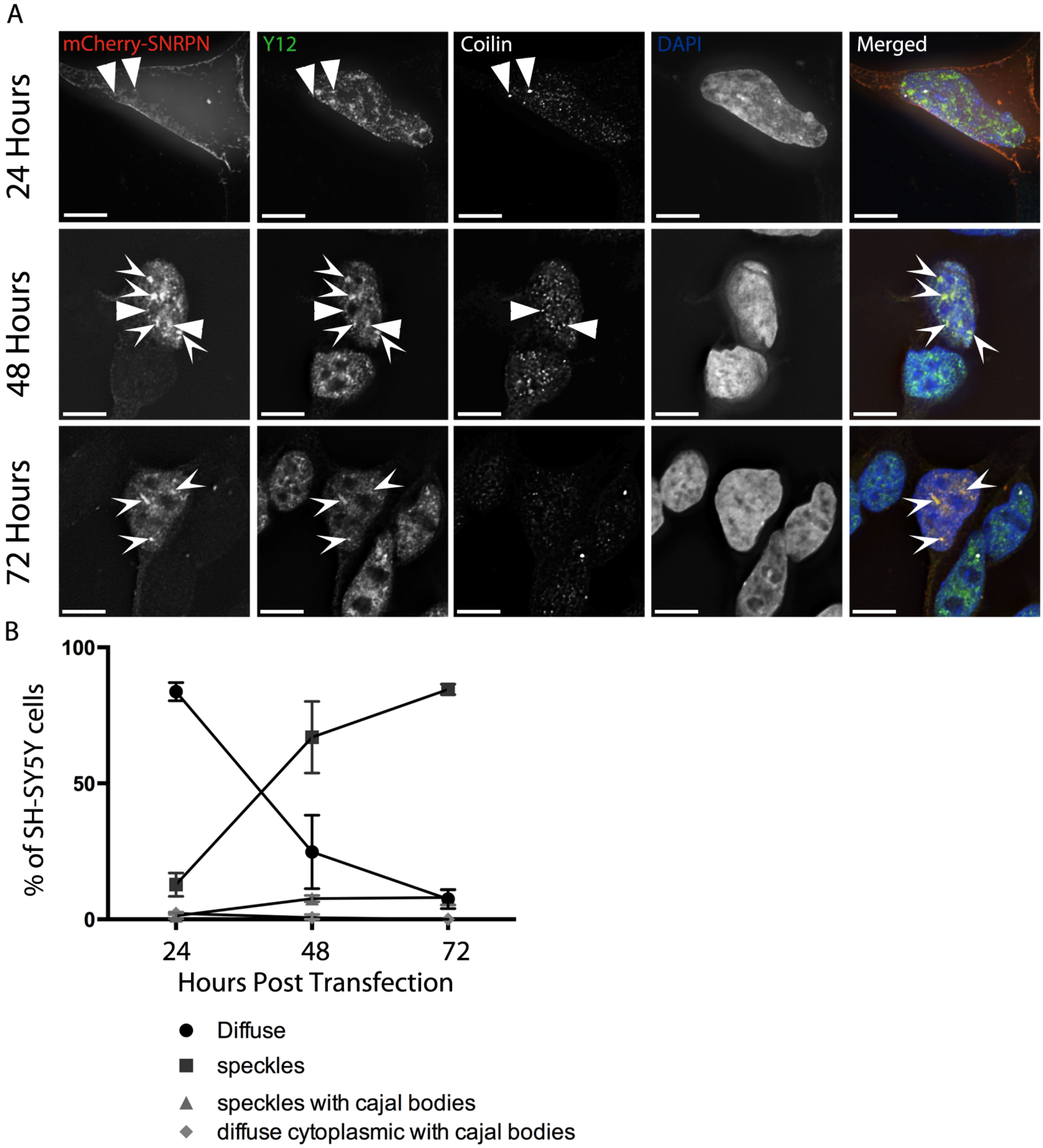

Figure S1: mCherry-SNRPN exhibits similar behaviour to both YFP-SNRPN and SNRPB in SH-SY5Y cells. A) SH-SY5Y cells transiently expressing mCherry-SNRPN and fixed after 24, 48 and 72 hours show variations in distribution of the mCherry-SNRPN with time. Immunostaining with Y12 (green on overlay) and anti-coilin (white on overlay) shows splicing speckles (arrowheads) and Cajal Bodies (CBs, triangles) respectively. Images are deconvolved z-stacks with 0.2 μm spacing. Bar=7μm. B) mCherry-SNRPN initially localises diffusely in the cytoplasm, before localising to speckles at the 48 and 72 hour time-points. 3 independent experiments, n=100 cells per experiment. Data shown is mean ± SD.

Journal of Cell Science • Supplementary information

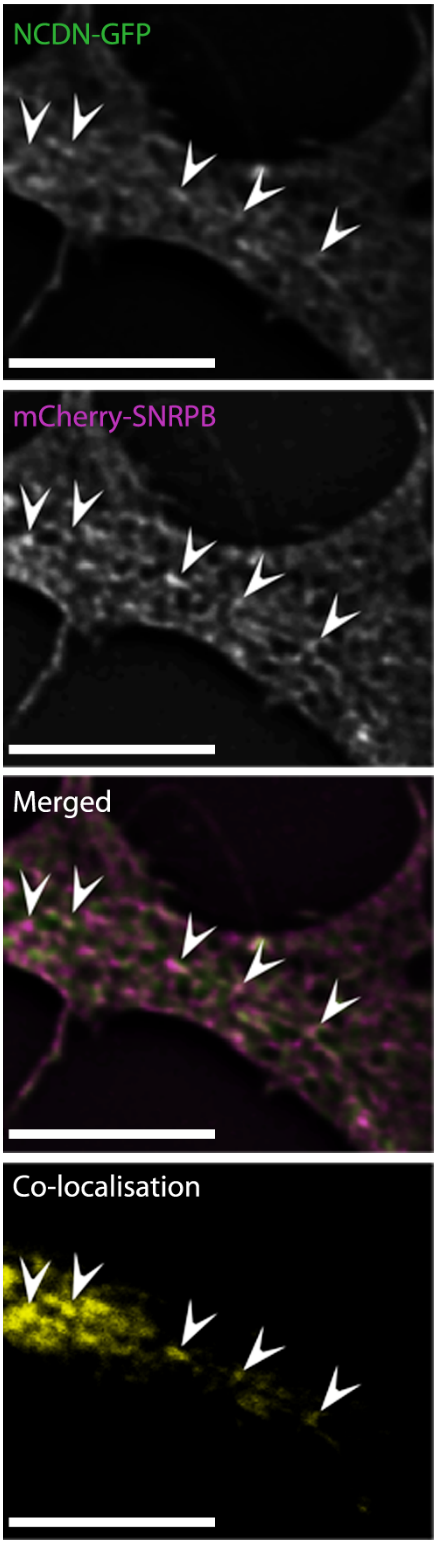

Figure S2: NCDN and SNRPB co-localise in neurites of SH-SY5Y cells constitutively expressing mCherry-SNRPB, and transiently expressing NCDN-GFP. Arrows identify SNRPB containing-vesicles with NCDN co-localisation. Bar= 7μm, images are single deconvolved z-sections. Co-localisation images were generated in Volocity, using automatic thresholds on non-deconvolved z-sections (see materials and methods).

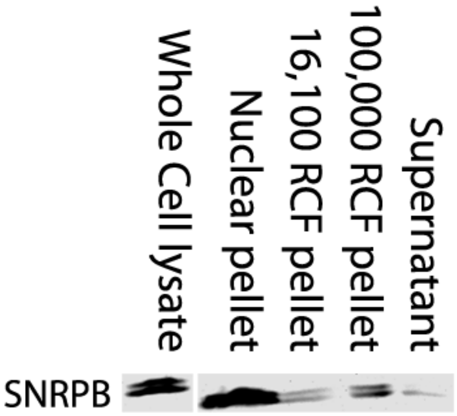

Figure S3: Endogenous SNRPB fractionates similarly to YFP-SNRPB and YFP-SNRPN, present in the 100,000 RCF vesicle fraction in and highly enriched in the nuclear pellet of fractionated SH-SY5Y cells. The gap between bands in the whole cell lysate and cell fractions signifies omitted lanes.

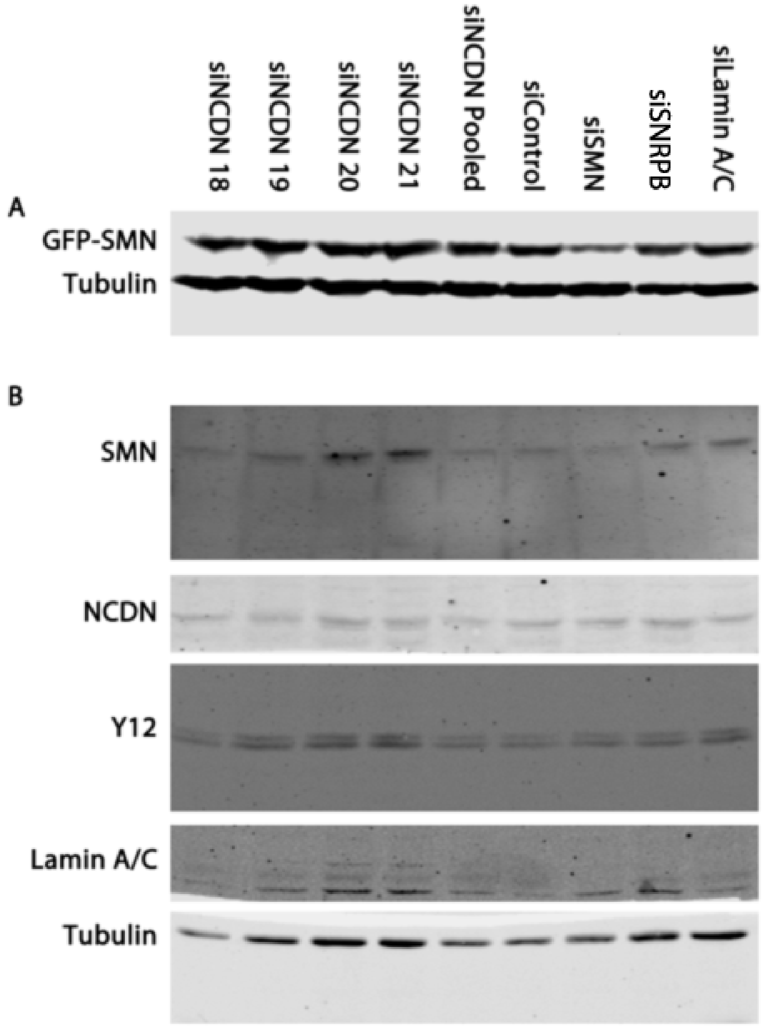

Figure S4: Quantified immunoblots of proteins targeted by siRNA in SH-SY5Y cells from Fig 5C. Equal volumes of lysate were immunoblotted, with band intensity of A) GFP-SMN, and B) SMN, NCDN, SNRPB (Y12) and lamin A/C quantified, and normalised to band intensity of tubulin, to determine the relative levels of protein expression.

Journal of Cell Science • Supplementary information

Identified protein	Accession number	Molecular Weight	SNRPN	SNRPB	YFP
small nuclear ribonucleoprotein polypeptide B * [Homo sapiens]	gi|53690154	24 kDa	13	11	2
small nuclear ribonucleoprotein Sm D2 isoform 1 [Homo sapiens]	gi|4759158	14 kDa	10	8	4
small nuclear ribonucleoprotein Sm D1 [Homo sapiens]	gi|5902102	13 kDa	5	5	4
small nuclear ribonucleoprotein E [Homo sapiens]	gi|4507129	11 kDa	5	4	2
small nuclear ribonucleoprotein F [Homo sapiens]	gi|4507131	10 kDa	5	3	0
small nuclear ribonucleoprotein Sm D3 [Homo sapiens]	gi|4759160	14 kDa	4	4	3
small nuclear ribonucleoprotein G [Homo sapiens]	gi|4507133	8 kDa	4	3	0
survival motor neuron protein isoform a [Homo sapiens]	gi|13259527	30 kDa	8	7	0
GEMIN5 protein [Homo sapiens]	gi|219517971	168 kDa	22	25	0
HC56 (Gemin 4) [Homo sapiens]	gi|10945430	119 kDa	18	11	0
DEAD box RNA helicase Gemin3 [Homo sapiens]	gi|6503002	92 kDa	12	18	0
gem-associated protein 2 isoform alpha [Homo sapiens]	gi|4506961	32 kDa	6	4	0
gem-associated protein 8 [Homo sapiens]	gi|8923481	29 kDa	6	2	0
Gem (nuclear organelle) associated protein 6 [Homo sapiens]	gi|17390437	19 kDa	3	3	0
gem-associated protein 7 [Homo sapiens]	gi|13376001	15 kDa	3	2	0
WD-40 repeat protein (Unrip) [Homo sapiens]	gi|4519417	38 kDa	16	16	0
U6 snRNA-associated Sm-like protein LSm4 isoform 1 [Homo sapiens]	gi|6912486	15 kDa	5	4	0
U6 snRNA-associated Sm-like protein LSm2 [Homo sapiens]	gi|10863977	11 kDa	4	4	0
U6 snRNA-associated Sm-like protein LSm6 [Homo sapiens]	gi|5901998	9 kDa	2	2	0
U6 snRNA-associated Sm-like protein LSm3 [Homo sapiens]	gi|7657315	12 kDa	2	2	0
U6 snRNA-associated Sm-like protein LSm7 [Homo sapiens]	gi|7706423	12 kDa	2	0	0
U7 snRNA-associated Sm-like protein LSm11 [Homo sapiens]	gi|27735089	40 kDa	2	0	0
protein arginine N-methyltransferase 5 isoform a [Homo sapiens]	gi|20070220	73 kDa	30	31	3
methylosome protein 50 [Homo sapiens]	gi|13129110	37 kDa	12	10	0
methylosome subunit pICln [Homo sapiens]	gi|4502891	26 kDa	7	8	0
CD2 antigen cytoplasmic tail-binding protein 2 [Homo sapiens]	gi|5174409	38 kDa	13	12	0
PERQ amino acid-rich with GYF domain-containing protein 2 isoform c [Homo sapiens]	gi|156766047	149 kDa	2	4	0
WW domain-binding protein 4 [Homo sapiens]	gi|6005948	43 kDa	3	0	0
formin binding protein 4, isoform CRA_a [Homo sapiens]	gi|119588290	90 kDa	2	2	0
neurochondrin isoform 2 [Homo sapiens]	gi|62526031	79 kDa	5	0	0
nuclear receptor coactivator 6 interacting protein, isoform CRA_b [Homo sapiens]	gi|119607168	97 kDa	13	0	0
7SK snRNA methylphosphate capping enzyme isoform A [Homo sapiens]	gi|47271406	74 kDa	13	0	0
RNA-binding protein 40 [Homo sapiens]	gi|40538732	59 kDa	13	0	0

Table S1: A selected dataset from the interactome analysis of SNRPN and SNRPB confirms efficient identification of known Sm protein interactors. All other core Sm family proteins were identified in the interactome analysis, as well as SMN, all Gemin components of the SMN complex and several LSm proteins, including LSm11 (found only in the U7 snRNP). Additionally, several members of the methylosome, where SmB post-translational modifications occur, were identified including Protein arginine N-methyltransferase 5 (PRMT5). Several previously identified SNRPB interactors (CD2 antigen cytoplasmic tail binding protein-2, PERQ2, WW domain-binding protein 4 and Formin binding protein 4) (Bedford et al. 1998, Bedford et al. 2000, Kofler et al. 2004, Kofler et al. 2005) were identified to interact with at least one of the proteins. * denotes that spectra from SNRPN were pooled with those from SNRPB by Scaffold due to sequence similarity in the majority of the proteins. Neurochondrin, and other proteins discussed are also shown here. Values are number of unique peptides identified.

Journal of Cell Science • Supplementary information

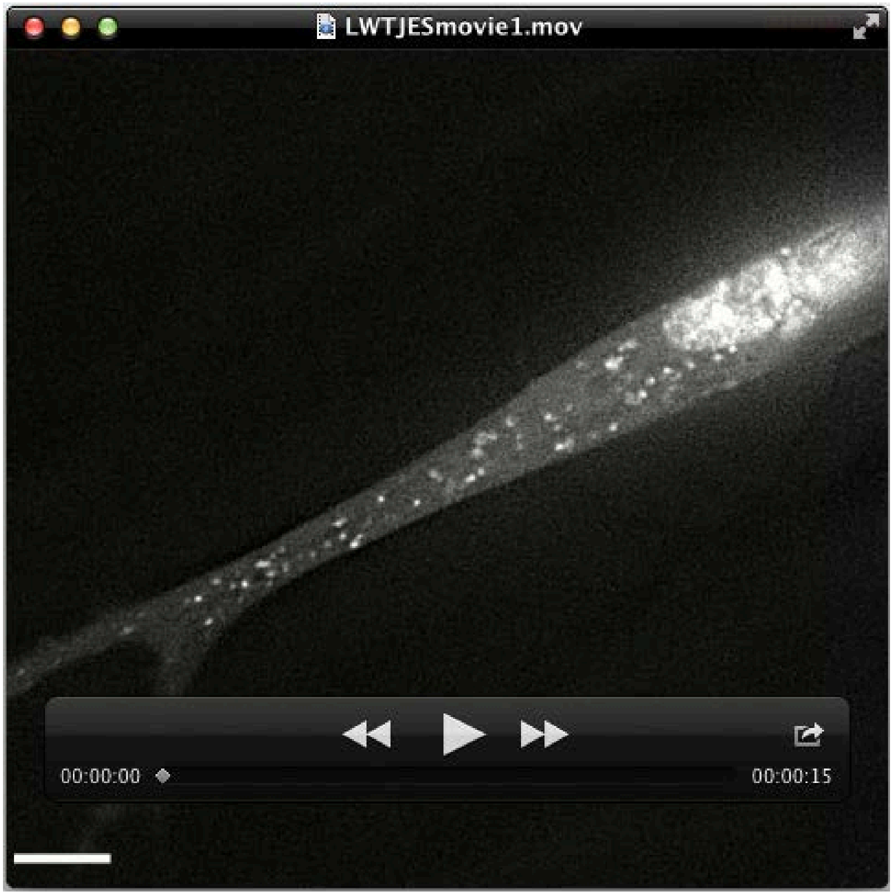

Movie 1: punctate structures containing mCherry-SNRPN within the cytoplasm of SH-SH5Y cells are mobile. Images were taken every ~2 seconds for ~150 seconds. Movie is a projection of 3 deconvolved z-stacks taken with 0.5μm spacing. Bar=7μm.
